# Bioelectrical synchronization of *Picea abies* during a solar eclipse

**DOI:** 10.1098/rsos.241786

**Published:** 2025-04-30

**Authors:** Alessandro Chiolerio, Monica Gagliano, Silvio Pilia, Paolo Pilia, Giuseppe Vitiello, Mohammad Dehshibi, Andrew Adamatzky

**Affiliations:** ^1^ Bioinspired Soft Robotics, Istituto Italiano di Tecnologia, Genova, Italy; ^2^ Unconventional Computing Laboratory, University of the West of England, Bristol, UK; ^3^ Cyberforest Experiment, Paneveggio (TN), Italy; ^4^ Biological Intelligence Lab, Southern Cross University, Lismore, New South Wales, Australia; ^5^ OpenAzienda S.r.l., Macomer (NU), Italy; ^6^ Dipartimento di Fisica `E.R. Caianiello', Università di Salerno, Fisciano (SA), Italy; ^7^ Departamento de Informàtica, Universidad Carlos III de Madrid, Leganés, Spain

**Keywords:** synchronization, spruce, cyberforest, quantum field theory, fractal dimension, entropy

## Abstract

Regular light–dark cycles greatly affect organisms, and events like eclipses induce distinctive physiological and behavioural shifts. While well documented in animals, plant behaviour during eclipses remains largely unexplored. Here, we monitored multiple spruce trees to assess their individual and collective bioelectrical responses to a solar eclipse. Trees anticipated the eclipse, synchronizing their bioelectrical behaviour hours in advance. Older trees displayed greater anticipatory behaviour with early time-asymmetry and entropy increases. These results reveal a relationship between trees, shaped by individual age and physiology as well as collective history. This highlights the significance of synchrony in plants, offering new insights into coordinated behaviours in nature.

## 1. Introduction

Sunlight and its periodicity drive global weather patterns, seasons and climate and make life possible on our planet. Daily and seasonal cycles of natural light organize biological systems by synchronizing their internal clock with the geophysical cycles of the Earth [1,2].

At a time marked by growing human-induced changes to natural cycles, unusual astronomical events such as eclipses effectively function as natural experiments [3], providing valuable insights into how living organisms respond to sudden and infrequent changes in their environment [4]. Even with their infrequent and momentary occurrence, eclipses and their impact on organismal behaviour have been observed for millennia [5] and are now well documented. In humans, for example, solar eclipses have played a transformative role inspiring awe and arousing social cohesion and prosocial tendencies, even capable of ending wars [6]. Heightened tendencies of individuals to form collectives by huddling, gathering and synchronizing group movements during solar eclipses are also seen across several terrestrial and aquatic animal groups [7]. These coordinated behaviours during eclipses suggest a potential role in enhancing survival chances for individuals and their groups. While these events may not pose an immediate threat to survival, they elicit adaptive responses that reflect broader strategies for coping with sudden and unpredictable environmental changes. Such behaviours likely evolved as mechanisms to reduce individual vulnerability by enhancing group vigilance and coordination in uncertain situations [8]. These responses can increase the resilience of both individuals and groups to environmental perturbations. If solar eclipses play such a vital role in shaping individuals and their groups to ensure collective survival across species, it is remarkable that very little is known about how plants respond to these astronomical events and such knowledge is limited to responses at the individual level [9–15], overlooking that group behaviour is also observed in plants [16,17].

**Figure 1.**
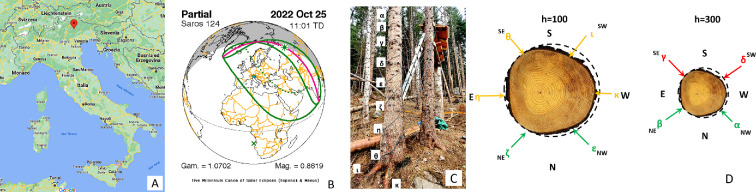
Experimental set-up to simultaneously monitor the electrome of multiple trees during solar eclipse. (A) The location of the experimental site at the Costa Bocche forest near Paneveggio in the Dolomites area, Italy. (B) Diagram of the Saros 124 event, the solar eclipse that occurred on 25 October 2022. Green continuous lines trace the Sun's shadow at ground level; the dashed line corresponds to a coverage of 50% of the Sun's disc and the pink line to the Sun's path. Eclipse predictions by Fred Espenak, NASA's GSFC. (C) *In situ* installation of 10 xylematic electrodes (labelled with Greek letters) on a spruce tree. Stainless steel threaded rods of 6 mm diameter were spaced 50 cm apart along the trunk and in contact with the core of the tree. Each electrode was then connected to the CyberTree devices via low-impedance audio cables. (D) Horizontal view of the phloematic configuration with electrodes in contact with the superficial layer of the tree and radially arranged in two arrays: (i) one array was located at 1 m above ground with electrodes at a radial distance of 60° from each other; and (ii) the other array was located at 3 m above ground with electrodes at a radial distance of 90° from each other. Cardinal and ordinal directions are indicated.

In this study, we leverage our newly developed remote measurement system [18] to simultaneously monitor multiple trees in a forest. This allows us to directly test whether and to what extent individual trees respond to a solar eclipse together, functioning as a larger living collective. We investigated the electrical signals (electrome) [19] of spruce trees (*Picea abies*) to characterize their bioelectrical activity during a partial solar eclipse that occurred in a forest located in the Dolomites mountain region, northeastern Italy (figure 1A,B).

The word electrome refers to the collection of electrical activities generated by living cells or tissues in an organism, encompassing all bioelectrical signals, such as action potentials, ion channel activities and electrical potentials across membranes [19]. It is analogous to terms like ‘genome’ or ‘proteome’, but specifically focuses on the electrical properties and phenomena within biological systems. A biological signal like those within the electrome is an active, functional electrical or biochemical process that originates within an organism and is used for communication or coordination within the organism’s body. Biological signals are a subset of the electrome ensemble. Electrome dynamics enable plants to coordinate various physiological functions for rapid response to environmental changes [20–23]. These dynamics exhibit non-random behaviour, long-range temporal correlations and persistence [24], suggesting a potential role in long-distance signalling [20,25,26]. The studies by Saraiva *et al*. [24] and Souza *et al*. [20], which focused on electrical signalling in individual plants under conditions like osmotic stimuli and temperature variations, provide foundational insights into the plant electrome dynamics. These insights suggest that trees may coordinate physiological responses to environmental changes, including unique disturbances like those caused by a solar eclipse. This supports our hypothesis that by monitoring electrome changes during the eclipse, we can identify distinct stages of a coordinated behavioural response. In a previous study within this forest, we established methods for measuring, analysing and sorting electrome dynamics of spruce trees at different stages (healthy young, healthy old, logs) and found correlations with solar (and lunar) cycles [18], while other studies related the electrome of pine trees to meteorological and geomagnetic parameters [27]. Building on this knowledge, we hypothesize that by monitoring electrome changes during the eclipse, we can identify the distinct stages of a behavioural response to a specific event, like a solar eclipse.

## 2. Material and methods

### 2.1. Electrome measurements

We investigated the bioelectrical response of spruce trees to a partial solar eclipse using a network of custom-built, low-cost, low-power and low-temperature-resistant CyberTree devices. These features enabled remote, continuous data acquisition during the eclipse event (details in electronic supplementary materials, tables S1–S3 and figures S1–S7). The CyberTree devices enabled us to simultaneously monitor bioelectrical activity from multiple trees (n=3 healthy trees and n=5 tree stumps) across four sites (D, E, F and G) within the same forest (figure 1C). Specifically, we compared the bioelectrical activity of:

— Two healthy, ∼70-year-old trees: one in full sun (site D) and one in full shade (site F).
— One healthy, ∼20-year-old tree in full shade (site G).
— Five stumps of old trees, originally part of a pristine forest at site E, which were devastated by the storm Vaia in 2018.

For each tree, we measured bioelectrical potentials using five pairs of differential electrodes. Each electrode pair was connected to a differential amplifier before data recording (details in electronic supplementary material, section S2). We labelled these electrodes with Greek letters (α, β, γ, δ, ϵ, η, ζ, θ, ι, κ). This set-up, with electrodes placed on both the inner (xylem) and outer (phloem) layers of the tree, allowed us to capture information about bioelectrical activity throughout the tree’s vascular system (figure 1D). For the collection of xylematic bioelectrical potentials from one tree, 10 stainless steel (AISI 316) threaded rods of 6 mm diameter were utilized. These rods were evenly spaced along the trunk, uncovered portions of roots and logs, with a separation distance of 50 cm. Each tree was carefully measured and the centre of the circumference calculated every 50 cm from ground level to the highest electrode level. Holes were realized intercepting the centre of the tree at every level (ranging from a minimum of 16 up to a maximum of 60 cm). Stainless steel rods were cut at exactly the radius of their respective level, labelled, insulated with the exception of the terminal portion and inserted into their specific holes. Similarly, electrodes in the phloem were inserted in much more shallow holes (2 cm). Trees were connected either with the xylematic electrodes or with the phloematic ones. Each electrode pair was connected to a differential amplifier prior to recording the data. For the collection of phloematic bioelectrical potentials, two recording sets of electrodes were positioned around the trunk of another tree. One set was located 1 m above the ground level with a radial distance between the electrodes of 60°, while the other was located 3 m above the ground level with a radial distance of 90°. As with the xylematic potential collection, each electrode pair was connected to a differential amplifier prior to analogue to digital conversion. The same threaded rods were used to collect bioelectrical signals from five logs. The first rod was inserted at the top of each log, and the second was positioned along one of the roots, 50 cm away from the first. Both rods were then connected to the differential amplifier. Signals from the trees to the differential amplifiers were transmitted using a double-shielded ultra-low capacitance INCA1050HPLC cable from MD Italy, designed for high-fidelity audio applications. Cables were connected to rods using soldered terminal lugs (copper made), mechanically screwed to the differential amplifiers terminals. The measured contact resistance component was 200 ± 50 mΩ, the cable resistance component was 50 ± 10 mΩ m^−1^ and the cable capacitance component was 150 ± 10 pF m^−1^.

### 2.2. Complexity measures

To sufficiently characterize the nonlinear, non-Gaussian behaviour of the bioelectric signals in trees, which are complex biological systems, we employed a combination of entropy, diversity, expressiveness, complexity and fractal measures. These higher-order complexity measures reduce uncertainty and enable better characterization of the inherent signal properties and system dynamics [18,28,29]. The following information-theoretic complexity measures were computed on the raw bioelectric potential data within 10 min sliding windows.

1. The Shannon entropy ($I_{1}$) [30] quantifies the uncertainty and information content in the signal. Higher entropy indicates more unpredictability. Given a random variable s with n elements, $s=\{s_{1},s_{2},\cdots ,s_{n}\}$, and its probability distribution $p(s)=\{p(s_{1}),p(s_{2}),\cdots ,p(s_{n})\}$, the Shannon entropy is given by
(2.1) $$I_{1}=-\sum_{i=1}^{n}p(s_{i})\;\log\;\left(p(s_{i})\right).$$
2. The Rényi entropy ($I_{2}$) [31] and Tsallis entropy ($I_{3}$) [32] measure signal diversity, capturing higher-order moments of the probability distribution compared to Shannon entropy. The Rényi entropy, with parameter q=2, is
(2.2) $$I_{2}=\frac{1}{1-q}\;\left(\ln\;\left(\sum_{i=1}^{n}p(s_{i}{)}^{q}\right)\right).$$
  The Tsallis entropy, with non-extensivity parameter q=2 and constant k=1, is:
(2.3) $$I_{3}=\frac{k}{1-q}\;\left(1-\sum_{i=1}^{n}p(s_{i}{)}^{q}\right).$$
3. The space filling ($I_{4}$) [33] represents the signal sparsity as the fraction of non-zero values. It indicates signal bursts and overall activity level.
4. The expressiveness ($I_{5}$) [34] is the ratio of Shannon entropy to space filling. It quantifies the efficiency or ‘economy’ of signal diversity.
5. The diversity index ($I_{6}$) [35] measures the richness and evenness of signal patterns, considering the number of unique activity types. It is key for assessing shifts in signal dynamics. With parameter q=3:
(2.4) $$I_{6}=\frac{1}{\sqrt[{q-1}]{\sum_{i=1}^{n}p(s_{i})p(s_{i}{)}^{q-1}}}={\left(\sum_{i=1}^{n}p(s_{i}{)}^{q}\right)}^{1/1-q}.$$
6. The Simpson diversity ($I_{7}$) [35] also measures concentration and diversity of signal types: $I_{7}=\sum_{i=1}^{n}p(s_{i}{)}^{2}$. It ranges from 0 to 1, with 1 indicating infinite diversity.
7. The Lempel–Ziv complexity ($I_{8}$) [36] assesses temporal diversity and complexity by estimating signal compressibility. It is sensitive to changes in the underlying physiological states.
8. The perturbation complexity index (PCI) ($I_{9}$) [37] normalizes the Lempel–Ziv complexity of the spatiotemporal pattern with respect to the Shannon entropy ($I_{1}$).
9. The Kolmogorov complexity ($I_{10}$) [38] calculates the minimum description length or compressibility of the signal, measuring its algorithmic complexity:
(2.5) $$I_{10}=\min\{|P||P\in {0,1}^{*},U(P)=s\},$$
  where U(P) is the output of the binary program P when executed on a fixed reference universal Turing machine and |P| is the program length.
10. The fractal dimension ($I_{11}$) [39] quantifies the self-similarity and scaling properties of the signal across time, useful for identifying transitions in system dynamics. Using the Higuchi method, it is calculated as $I_{11}=\log(n)/\log(1/d)$, where d is the time step and n the signal length.

### 2.3. Evaluating synchronicity in distinct processes

In order to determine the precise timing when two measured processes are most synchronized, we calculate the cross-correlation function through equation (2.6) (adapted from [40]), where g represents the cross-correlation function, d and f denote two measured distinct processes (bioelectrical potentials), r symbolizes the Cartesian volume in the space where the physical processes are collected, t represents the time and $\tau =t+\delta_{d\to f}$ represents the delay $\delta_{d\to f}$ of the process f with respect to process d:


(2.6) $$g\left(d(r,t);\;f(r,\tau )\right)=\frac{\sum_{t}\{[d(r,t)-<d(r,t)>][f(r,\tau )-<f(r,\tau )>]\}}{\sqrt{\left\{\sum_{t}[d(r,t)-<d(r,t)>{]}^{2}\}\{\sum_{t}[f(r,\tau )-<f(r,\tau )>{]}^{2}\right\}}}.\;$$

### 2.4. Quantum field theory theoretical analysis

In our theoretical analysis we use the quantum field theory (QFT) formalism. The motivations and benefits are the following. Trees are *open*, and hence *dissipative*, systems, continuously exchanging (releasing and receiving) matter and energy in various forms with their environment. Moreover, they are *aging* systems, the origin in the time of their life cannot be moved and their time evolution (*the arrow of time*) cannot be inverted. All this means that there is ‘breakdown’ of the symmetry under time translation and time reversal, respectively, which is also reflected in equation (2.6).

The canonical (classical and quantum Hamiltonian) formalism, however, only applies to *closed*, non-dissipative systems. Use of the canonical formalism thus requires to satisfy the mathematical constraint of ‘closing’ the system, which is achieved by considering both the tree system and its environment; a well-known strategy adopted, e.g. in dealing with the time-irreversible evolution of thermodynamic systems. On the other hand, our aim is to describe the macroscopic behaviour of trees in terms of the dynamics at the microscopic level involving biomolecules, ions, electric dipoles of water molecules, lymph and other molecular structures. An *in materia* computing approach is necessary since trees express their multiple microscopic configurations as macroscopic behavioural properties [41]. In physics, the only available formalism able to deal with microscopic components is provided by quantum theories, which therefore we need to use.

Considering then the breakdown of time-translation and time-reversal symmetries, the tree’s dissipativity, mentioned above and the relevance of thermal effects, all of them implying the mathematical constraint of ‘closing’ the system, we adopt, as already done in [18,42], the thermal QFT formalism, called Thermo Field Dynamics (TFD) 3313 [43,44], where the tree’s degrees of freedom $a_{k}$ are indeed introduced together with the environment ones, ${\tilde{a}}_{k}$. The couples $\left(a_{k},{\tilde{a}}_{k}\right)$, for any k, describe then the coupled system {tree-system, environment}, which is ‘closed’ provided that the energy in all of its possible forms out-going from trees is in-going into the environment, and vice versa*.*

Apart from these motivations and mathematical constraints, which ones are the benefits of using QFT? One of them is that the QFT analysis is not simply a phenomenological one; as said, it concerns the underlying biomolecular and bioelectric dynamics, thus giving access to the understanding of the deeper level of molecular activity. In addition, the QFT state of the couples $\left(a_{k},{\tilde{a}}_{k}\right)$ turns out to be a coherent state (given by electronic supplementary material, equation (S6)) [45]; a new result by itself valuable from the physical standpoint of plant studies, out of reach in other approaches. Moreover, this fact allows the comprehension of the mechanism leading from the microscopic molecular dynamics to its manifestations at the macroscopic level, also recovering thermodynamic quantities such as heat, entropy, free energy, etc. As shown in [18,42], the QFT analysis also gives the possibility to quantify the reaction (opposition) of the tree to variations of the environment temperature, aimed to protect the state of the tree against temperature changes; thus providing the derivation of the Le Chatelier principle [46,47] which is not obtained in phenomenological approaches.

A further result of the QFT analysis is that a dynamic correlation (entanglement) exists among trees, coming as a phase synchronization, not based on matter exchanges, e.g. through fluids and molecular exchanges via roots, or by air currents, etc. This is a new result reported in [18,42], typical of dissipative dynamics, out of reach in other approaches, opening on a solid scientific ground a new understanding of the *forest*, moving away from the picture of a collection of trees, approaching instead the picture of a community of phase correlated individuals, collectively playing as in an orchestra in synchrony with the ecosystem’s life.

Finally, the entanglement accounts for the non-vanishing value of the cross-correlation function [18] in equation (2.6), which may be thus considered as its physical manifestation, not solely of the eclipse phenomenon. The degree of entanglement is given by the value of the covariance of the state (electronic supplementary material, section S5).

## 3. Results

### 3.1. Individual tree responses to the solar eclipse

**Figure 2.**
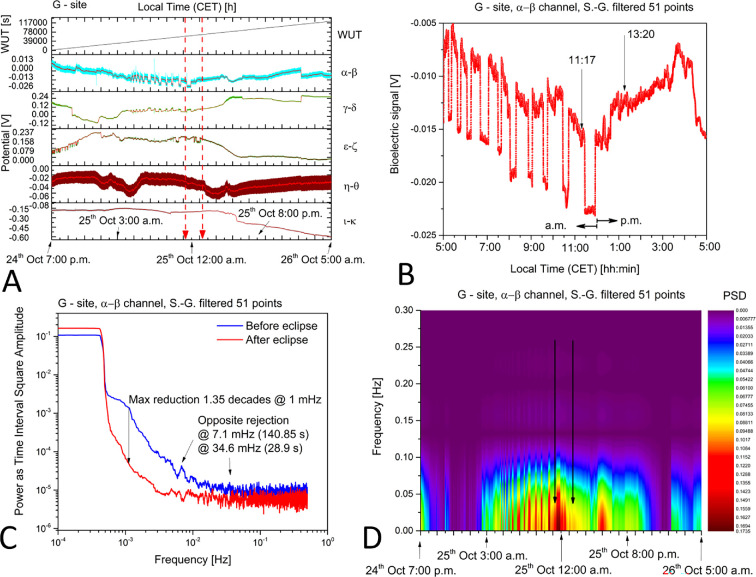
Bioelectrical potentials recorded from the 20-years-old tree at site G during the solar eclipse. (A) Raw measurements were collected from five differential channels. The data chunk spanned 34 h and featured the five differential channels (curves) plus the ‘wake-up time’ (WUT) of the CyberTree device as a reference to correlate with spikes that might occur during eventual restart. In this specific case, the WUT corresponds to the experiment duration. The two red arrowed broken lines indicate the starting (11.17) and the ending (13.20) of the solar eclipse. (B) Enlarged view of the α−β differential channel with solar eclipse timing indicated. A first-order Savitzki–Golay (S.-G.) function with 51 points was applied to smooth the data and allow the underlying oscillations and correlations between the five differential channels to become more noticeable. (C) FFT analysis of 40 000 s before the solar eclipse and 40 000 s after, taken from the α−β differential channel. S.-G. smoothing applied. As indicated, the noise power dropped by 1.35 decades at 1 mHz, and two peaks showing opposite rejection were found around 7 and 35 mHz, corresponding to periods of 141 and 29 s, respectively. (D) STFT analysis of the entire data shown in panel (A), limited to the α−β differential channel. The black arrows indicate the starting and ending of the solar eclipse. S.-G. smoothing applied. The experiment time is reported (CET time zone).

Based on the starting and ending point of the solar eclipse, we detected a general trend directly associated with the geometric occultation of the Sun and corresponding to an overall reduction of the bioelectrical potential at all sites with living trees. Onsite climatic recordings of temperature, relative humidity, rainfall and daily solar radiation are shown in electronic supplementary material, section S4. During the evolution of the eclipse there was a temperature increase between 5.9°C and 8.8°C, a reduction of the relative humidity between 91% and 82%, no rainfall and no evidence of solar radiation changes in the daily average. At the shaded site G, for example, the young 20-years-old tree exhibited a reduction of its bioelectrical potential, evident particularly for the electrode pairs ϵ−ζ and η−θ (figure 2A). Remarkably, we detected a sharp spiking activity, particularly at the levels of the two upper pairs α−β and γ−δ and visible as several quasi-square-waves concentrating until the onset of the eclipse, and then disappearing in the stage immediately following the eclipse end (figure 2B–D). The FFT and STFT analyses shown in the last two panels visualize the differences between minutes before and minutes after the eclipse. Fourier analysis was preferred to other techniques for the following reasons: (i) rapid analysis over the entire frequency domain; (ii) efficient calculations that do not draw too much energy, in view of a direct implementation on the remote CyberTree devices; and (iii) well established tools, easily found. The non-stationary nature of the electrome we have measured is easily recovered by performing Fourier analysis over short time domains and comparing them (as in figure 2C,D).

**Figure 3.**
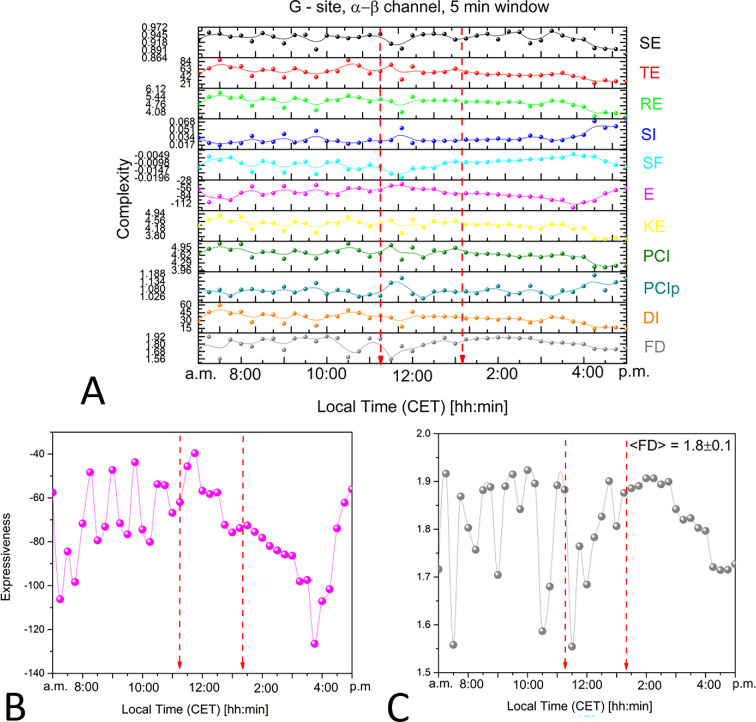
Complexity analysis of bioelectrical potentials recorded from the 20-years-old tree at site G during the solar eclipse (indicated by the two red arrowed broken lines). (A) Comparison of all the complexity measures using data chunks of 5 min: Shannon entropy, Tsallis entropy, Renyi entropy, Simpson index, space-filling, expressiveness, Kolmogorov entropy, perturbation complexity index, permuted perturbation complexity index, diversity index and fractal dimension. The two red arrowed broken lines indicate the starting (11.17) and the ending (13.20) of the solar eclipse. Detailed variation for (B) the expressiveness and (C) the fractal dimension during the solar eclipse. The experiment time is reported (CET time zone).

A more in-depth analysis of the signals collected from channel α−β comparing all complexity measures allowed us to appreciate the noise and fluctuation of almost all functions before the eclipse and a strong reduction afterwards (figure 3). For example, the expressiveness stopped its 5/10 min oscillating behaviour at the beginning of the eclipse, went into a smoother period that lasted hours after the end of the event, and then recovered to the original absolute values (figure 3B). Similarly, the fractal index showed a strong oscillation until the beginning of the eclipse, then a transition to a smoother area (figure 3C), indicating that the bioelectrical signal from the tree lost the natural variability in spiking and oscillations, which is observed as spontaneous electrical activity during a normal day.

At sites D and F, where we monitored the older trees, we also observed a reduction of the bioelectrical potential corresponding to the Sun occlusion (electronic supplementary material, figure S7). On the F-site tree, this reduction was particularly sharp at the top electrode pair γ−δ, but also detected at the level of the three bottom electrode pairs ϵ−ζ, η−θ and ι−κ, though taking place over a slightly broader timescale (electronic supplementary material, figure S7E). The D-site tree shows a bioelectrical potential discontinuity, matching well the timing of the eclipse, on at least two electrode pairs, specifically the top one α−β and the bottom electrode pair ι−κ (electronic supplementary material, figure S7A). In these older trees, we detected a slow change in the initial bioelectrical potential level by approximately 300% occurring during the first 50 000 s (≈ 13.8 h ≃ 14 h) (electronic supplementary material, figure S7E). For the F-site tree, for example, this trend was evident in the bottom electrode pairs ϵ−ζ, η−θ and ι−κ, where the pair facing the Sun and oriented southwards (ϵ−ζ) detected increased bioelectrical potentials and higher noise level. In contrast, the other two pairs detected a reduction. This trend was not found in the top pairs α−β and γ−δ. From the end of the eclipse until 120 000 s (≈ 3.3 h ≃ 3 h), all electrode pairs on the F-site tree detected a renormalization of the bioelectrical potential levels approaching their initial values. Finally, we detected less pronounced variations in the bioelectrical responses of the five tree stumps at the E-site (electronic supplementary material, figure S7C). However, a deeper analysis based on FFT tools revealed a clear difference in the biopotential spectrum before and after the eclipse. We see that the oscillation frequency increases after the astronomical event. The xylematic electrodes being connected more to the inner physiology of the plant and to the movement of fluids inside the trunks triggered by capillarity and water pressure in the soil, we deduce that a higher frequency in the oscillation of liquids is generated. Oscillating masses reduce their fluctuation periods when the mass increases; therefore, it is likely that the effective mass has increased.

### 3.2. Collective responses to the solar eclipse

**Figure 4.**
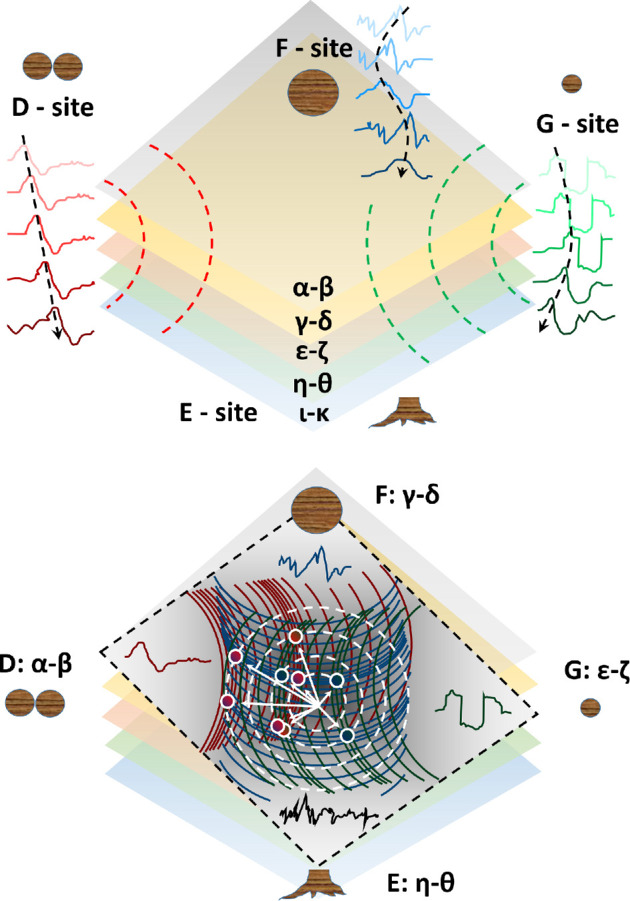
Bioelectrical potential recordings as stochastic processes in a multi-layered spatiotemporal arrangement. Top: each corner of the square corresponds to a recording site (from left to bottom, in a clockwise direction: D-site, F-site, G-site and E-site). Each set of recordings shows bioelectrical waves that propagate along the layers. Bottom: interference of stochastic processes showing constructive/destructive profiles against a common barycentre (white arrows). Red waves propagate from the D-site, blue waves from the F-site and green waves from the G-site. Constructive interference is pinpointed by white discs, whose inner colour gradient shows the fusion of the two originating processes.

To further investigate whether individual trees responded to the solar eclipse together as a collective system, we conducted an analysis of stochastic processes in a multi-layered spatiotemporal arrangement (figure 4). Within each site, we observe the propagating bioelectrical waves, which can be attributed to various factors such as the movement of lymph carrying ionic currents (observed at D-site, where xylem electrodes are positioned along the trunk), circadian processes following the Sun’s orbit (seen at F-site, where phloem electrodes are arranged radially along the trunk), spontaneous spike-like processes (observed in the young tree located at the G-site) or even noise from recording logs (figure 4A, top). Each stochastic process interacted with the others, akin to waves on the surface of water, exhibiting a certain level of temporal correlation (figure 4, bottom).

**Figure 5.**
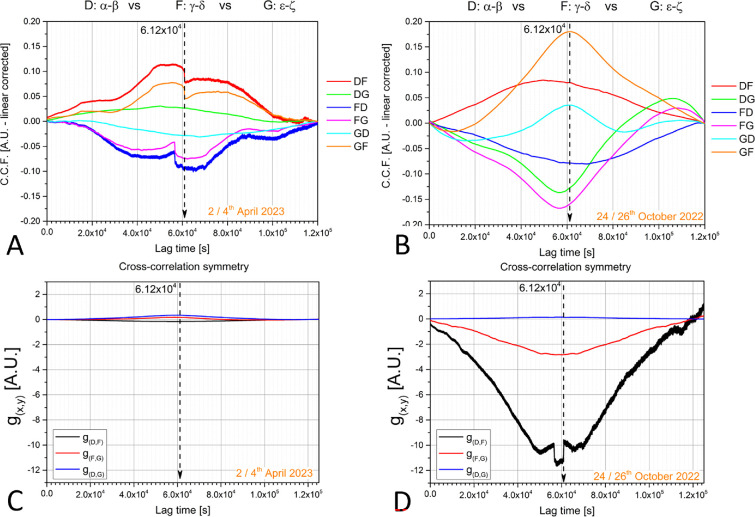
Cross-correlation functions calculated by comparing couple-wise processes occurring at the D-, F- and G-sites. (A) Cross-correlation functions calculated during a normal day. The choice of signals was totally random, comparing differential channels without any prior logic. Midday corresponds to $6\times 10^{4}$ s. (B) Cross-correlation functions are calculated during the solar eclipse. (C) Difference between direct and inverse cross-correlations during a normal day and (D) during the solar eclipse. Note that the lag analysis is run by ‘artificially scanning the time scale’, and although seconds of the analysis are equivalent to seconds of reality, the real time of the day cannot be reported on the *x*-axis.

The cross-correlation functions calculated during a normal day exhibited a degree of symmetry with an absolute variation of around 0.1 units (figure 5A). However, the degree of symmetry changed during the solar eclipse; absolute variations doubled in magnitude and had a smoother shape (figure 5B). In other words, during a normal day, there was little difference between any two instants in the bioelectrical propagations occurring between two trees. During the eclipse, however, any two generic instants were indeed very different from each other. This interference was particularly noticeable between the trees D and F, which is unsurprising given that, because of their age, older trees serve as hubs that boast a more dense network of connections within a forest [48].

The effects of the solar eclipse could also be monitored by calculating the difference between direct and inverse cross-correlations; for example, by considering the time lags measured on D-site electrome taking F-site electrome as a reference (direct correlation) and by considering time lags measured on F-site electrome taking D-site electrome as a reference (inverse correlation), plotted as a black line in figure 5C,D. As formalized in equation (3.1), the differential function of implicit space–time variables h (‘cross-correlation symmetry’) is a measure of the symmetry of two processes and should be zero for symmetrical ones:


(3.1) h(d;f)=g(d;f)−g(f;d).

In this analysis, signal propagation is intended as a temporal process. Therefore, we investigated whether a predictable event like the circadian cycle dictated by the daily rising and setting of the Sun appears symmetric or ordinary when compared to a less frequent event such as the solar eclipse. By putting the two groups of complementary differences on the same scale, it is clear how the daily solar cycle produces very symmetric processes with deviation in time smaller than 1. When the forest experienced the solar eclipse, however, the effects were much more pronounced with a deviation from symmetry that could be greater than 10 with maximum values for the older trees (the D–F couple). Not only was the deviation from temporal asymmetry more pronounced in this couple of older trees, but the symmetry-breaking process (and the accompanying increase in entropy; [49]) described by the vertical offset from zero, also commences earlier (by 1 h) and concludes later than in couples with old and young trees (see the offset in the black curve, figure 5D). Finally, by comparing the time lags between simultaneous signals, we were able to trace a response to the eclipse approximately 14 h ahead from the actual occurrence of the event.

### 3.3. Theoretical analysis of collective bioelectrical activity

In the study of biological systems, Schrödinger noted in *What is life?* [50] that the ‘regularities only on average’ emerging from ‘statistical mechanisms’ are not enough to explain the ‘enigmatic biological stability’. He stressed that the ‘classical physicist expectation’ to explain it solely on a statistical basis, ‘far from being trivial, is wrong’ [50].

Statistical methods are *necessary* (essential) for understanding biochemical processes; however, they are *not sufficient* to fully explain the remarkable efficiency and functional stability of biological systems. Schrödinger suggests that it may be accounted for by the quantum formalism describing various components of these systems, such as molecules, ions, currents and molecular electrical dipoles giving rise to bioelectrical potentials. Therefore, as said in §2.4, we analyse in terms of QFT formalism the dynamics of biochemical and bioelectrical activity of trees.

The remarkable agreement between QFT mathematical formalism and cross-correlation functions in complex tree systems underscores the importance of dissipation, or ‘openness’, in the trees’ biochemical activity. Also, the variations observed during the eclipse event (figure 5A,B) are likely due to the perturbations induced by the eclipse on the microscopic state at the molecular and ionic level, largely dependent on the electric dipoles of the water molecules, resins, lymph molecular components and other biomolecules of the tree [18]. During the event, the observational data suggest that phase correlations are enhanced, which is mirrored by the bioelectrical potentials.

As trees are open systems and their evolution is ruled by dissipative dynamics [51], we expect non-symmetric reactions to perturbing agents by differently aged trees, especially in older trees, that have been connected for a longer time with the environment. As shown by equation (3.1), such an asymmetry is expressed in the non-commutative character of the two processes d and f (see figure 5C,D for other pairs of trees). The theoretical analysis shows that the anti-symmetric state is the lowest energy state and, therefore, the one with a higher probability of being actually realized, as observed in our measurements (see formal details at the end of electronic supplementary material, section S5).

In condensed matter physics, an external input, no matter how weak, can disrupt the symmetry of the system’s state. This phenomenon is named spontaneous breakdown of symmetry (SBS) and is known to occur along with the dynamical formation of long-range correlations among the system’s elements and coherent condensation of associated quanta (the Nambu Goldstone, NG, quanta) in the system’s ground state, giving rise to ordered patterns [43,44,52,53]. The term ‘coherent condensation’ indicates that these long-range correlations are ‘in phase’, allowing them to coexist in the ground state without mutual destructive interference. Ordered patterns are identified by an order parameter. The transition from the microscopic quantum level to the macroscopic one is achieved through dynamical processes, with coherent condensation playing a pivotal role in this transformation.

Coherent condensation is observed across a wide range of temperatures, from sodium chloride’s $804^{\circ }$C melting point to iron’s loss of magnetization at $770^{\circ }$C, niobium compounds’ superconductivity vanishing at $-153^{\circ }$C and $-252^{\circ }$C in copper and bismuth compounds. Coherence is also observed in photosynthesis at room temperature [54,55]. In biological systems, which are inherently non-equilibrium systems, there are continuous transitions between dynamical regimes, (phase transitions) characterized by different levels of coherent condensation [56,57].

Being largely dependent on fluctuations of the electrical dipoles of many biomolecules, tree microscopic dynamics is invariant under dipole rotational symmetry transformations. Thus, environmental changes and inputs, including events such as an eclipse, may induce the spontaneous breakdown of the tree’s molecular dipole rotational symmetry transformations. Thus, environmental changes and inputs, including events such as an eclipse, may induce the spontaneous breakdown of the tree’s molecular dipole rotational symmetry [57]. The molecular dipole fluctuations act as quantum variables, and the quanta associated with the long-range correlations, called dipole wave quanta (DWQ) in our case, coherently condense in the system ground state. In other words, we expect entropy S and the rate of loss in time of coherent correlations to decrease as the eclipse commences, subsequently reestablishing symmetry within the system after the event.

Grounded on such a theoretical basis, our modelling then accounts for the observed behaviour of the cross-correlation function, as already seen, and several other features (figure 5). For example, the electromagnetic (EM) vector potential A(x,t), and therefore the bioelectrical potentials derived from it, is affected by the coherent condensation of DWQ through the gauge transformation A(x,t)→A(x,t)+∇λ(x,t), where the Coulomb gauge ∇⋅A(x,t)=0 and the constraint $\nabla^{2}\lambda (x,t)=0$ are assumed. λ(x,t) is related to the DWQ condensation density and, therefore, to the external agent triggering the SBS.

The theoretical modelling thus describes a direct relationship between the measured bioelectrical potentials and environmental changes from atmospheric effects, including circadian cycles, and light and gravitational effects before, during and after the eclipse. It also accounts for the observed fractal dimension features. Alterations in the fractal dimensions are recognized as indicators of shifts in the underlying structure, dynamics and behaviour of a system, often signalling impending transitions (eg. [58–60])). It is known that an isomorphism exists between deformed coherent states and fractal self-similarity [44,61,62]. We observed a correlation between changes in the fractal dimensions and alterations (‘deformations’) in the condensation densities of coherent states, associated with transitions between different dynamical regimes of the tree system. For example, we observed a decrease in fractal dimension, which was indicative of heightened coherent condensation density, hence signalling a more coherent regime, compared to periods preceding and following the event (as illustrated in figure 3C). Specifically, we concluded that the solar eclipse produced an SBS two orders of magnitude greater and higher coherent condensation of DWQ compared to normal daily cycles. This condition was observed especially in older trees, where thicker xylematic tissues featuring richer molecular fluxes are present.

As observed in §2.4, trees and environment modes $a_{k}$ and ${\tilde{a}}_{k}$, respectively, are entangled modes. The physical meaning of the entanglement is that trees are components of the forest collective system, resulting from the large net of tree correlations, whose number $N_{k}(t)$ is one of the condensed DWQ. The number of configurations accessible by such a net determines the forest entropy S. In terms of variations in the time ${\dot{N}}_{k}(t)$, the free energy minimization dF=dU−(1/β)dS=0  gives [63]


(3.2) $$dU=\sum_{k}\hbar \;\omega_{k}\;{\dot{N}}_{k}(t)dt=\frac{1}{\beta }dS=dQ\;,$$

where U denotes the energy of the uncorrelated system’s components and $\beta =1/k_{B}T$ is the inverse temperature, with $k_{B}$ the Boltzmann constant.

We see that as entropy decreases (and ordering increases), the rate of loss in time of coherent correlations decreases. As energy gets stored in the ordering of the system (U decreases), dU→TdS, we observe a power loss in the spectral analysis as the eclipse starts and during the event (figure 2C), and until the subsequent normal regime is reached after the eclipse (dU←TdS). A similar process aimed to restore the system to the state prior to the perturbing action was also observed in the fractal dimension behaviour (figures 3C and 5). By comparing direct and time-reversed cross-correlation functions of the bioelectrical potentials, our modelling adequately corroborates our experimental findings, which support the vision of the forest as a correlated (collective) system. Although the environment significantly impacts the individual state and bioelectrical response of trees, their inter-connectivity, as evidenced by correlations and synchronized activity, suggests a more cohesive, organism-like response at the forest level. By virtue of synchronized individual responses, the forest reproduces the different stages of the individual’s responses to the changing conditions to respond to an event like the solar eclipse.

## 4. Discussion

### 4.1. Relationship between bioelectrical potentials and environmental changes

Our approach described a direct relationship between the measured bioelectrical potentials and environmental changes from atmospheric effects, including circadian cycles, light and gravitational effects before, during and after the eclipse. We used both direct and time-reversed cross-correlation functions of the bioelectrical potentials to analyse how the electrical signals within trees are related to each other over time, and if there are patterns where one signal predicts or influences another. Our modelling effectively confirmed our experimental findings and reinforced the concept of the forest as a correlated system. Specifically, while environmental factors notably influenced the condition and bioelectrical reactions of individual trees, their interconnectedness, evidenced by correlations and synchronized activity, suggested a cohesive, organism-like response at the forest scale. Through synchronized individual responses, the forest mirrored the diverse responses of individual trees to changing conditions, thus responding collectively to environmental changes and events such as the solar eclipse. Recent observations, such as those highlighted by Beverly *et al.* [14] and discussed in the context of the April 2024 total solar eclipse, further illustrate the profound impact of solar eclipses on plant behaviour. These findings underscore how rapid transitions from darkness to light during an eclipse can disrupt biochemical processes and circadian rhythms, resulting in altered plant behaviour for several hours post-eclipse. While these studies focus on individual plant responses, our study extends this understanding by examining the collective dynamics of multiple trees in a forest. By observing these collective responses, we provide additional insight into how forests might buffer or amplify the effects observed at the individual level, contributing to a more comprehensive understanding of the ecological impact of such transient celestial events. These dynamics ultimately influence the forest ecosystem’s resilience, biodiversity and overall functionality. Considering such a scenario, we presented an analysis within the QFT framework established by [18,51].

The variations observed during the eclipse event (as illustrated in figure 5A,B) are likely due to perturbations induced by the eclipse on the microscopic state at the molecular and ionic level. These perturbations depend on factors such as the electric dipoles of water molecules, resins, lymph molecular components and other biomolecules within the tree [18]. Our data showed enhanced phase correlations during the eclipse, indicating a more coherent coordination of the tree’s biochemical processes. The observations in phase correlations were mirrored in the bioelectrical potentials data, thus indicating consistent findings across different analyses.

Additionally, distinct transients in the correlation functions were observed on a normal day, around $5.7\times 10^{4}$ s (≈16 h) before noon, likely caused by regular environmental fluctuations such as temperature or humidity changes influencing bioelectrical activity. However, no such transients were visible during the eclipse, suggesting that the stronger influence of the eclipse may have synchronized or suppressed smaller fluctuations. This highlights the significant impact of the eclipse on bioelectrical activity and suggests further investigation into the interaction between regular abiotic factors and eclipse-driven changes.

The solar eclipse made a sharp contribution to the breakdown of symmetry, increasing coherence and more coordinated behaviour among individual trees in the forest system. This effect influenced communication channels among trees, particularly those with older trees with thicker xylem tissues and richer molecular fluxes. That such effect was especially noticeable between the older trees came as no surprise, given that older trees, serving as hubs within a forest, have been shown to possess a more complex network of connections, as demonstrated by [48].

These findings align with prior research highlighting the interconnectedness of trees within forest ecosystems, emphasizing the significance of resource-sharing among individual trees (e.g. [48,64–69]; but see also [70]). These studies have predominantly relied on isotopic labelling methods and periodic sampling to track element movement within forest systems, aiming to elucidate the interconnectedness among trees and their role in supporting collective ecosystem health and productivity. The novelty introduced here is that we devised a continuous monitoring system for remotely tracking the bioelectrical activity of trees, facilitating real-time observation of their dynamic responses within the forest system and their exchange of information about environmental conditions. This unique approach proved particularly effective in revealing unexpected phenomena among trees, including the synchronization of their bioelectrical status during events like the eclipse. Our study thus offers additional experimental support for the notion of non-trivial effects in biological systems (e.g. reviewed by [71–73]), extending the microscopic scales to larger-scale dynamics [74,75].

### 4.2. Bioelectrical synchronization of trees during a solar eclipse

Patterns of synchronous activity have been observed in almost every animal group studied [76]. Notably, synchronized behaviour fundamentally involves the synchronization of movements [77]. Hence, likely due to their sessile nature, behavioural synchronization in plants has remained unexplored (and we are not concerned with phenological synchronization on the well-known circadian or seasonal timescales, such as masting [78], flowering [79] or oscillatory motions of stems and leaves [80]). By monitoring the electrome of spruce trees in their natural environment during the eclipse, our study provides evidence that trees display synchronized alterations in their bioelectrical activity patterns. Prior to this synchronized behaviour, the trees exhibited anticipation, a critical feature of synchronization behaviour displayed by many animal species that makes synchrony distinguishable from repetitive reactions to repetitive stimulation [77]. Specifically, the trees anticipated the solar eclipse by changing their bioelectrical behaviour several hours before the actual onset of the celestial event, which could cause substantial perturbations, such as drops in sap flow rates within trees [11] and disruptions in their vital hydraulic connection between the soil and the atmosphere [81].

While solar eclipses may seem rare from a human perspective, they follow cycles, such as the Saros cycle, which can occur well within the lifespan of long-lived trees. This predictability suggests that solar eclipses, despite their infrequency, are not perceived as random events by trees. Instead, long-lived trees may have developed mechanisms to anticipate and respond to these events, similar to their responses to seasonal changes. Our findings support this hypothesis, as older trees exhibited greater anticipatory behaviour during the eclipse, possibly reflecting environmental patterns established over time. The synchronization and early time asymmetry observed in older trees reinforce the notion that rare but recurring events, like solar eclipses, are integrated into the biological dynamics of these organisms.

Crucially, organisms must be able to detect cues alerting them that circumstances have changed in value to ensure that the right response occurs at the right time through anticipatory behaviours [82]. The cues that generated the anticipatory behaviour observed in these trees remain to be determined. We exclude sunlight and air temperature alterations induced by the solar eclipse as potential cues because the tree anticipated the event about 14 h before its occurrence when both Sun and Moon were at their zenith on the other side of the Earth (i.e. on the Solomon Islands and off the coast of Vietnam, respectively). However, potential cues might have temporal patterns dependent on Sun–Moon–Earth orbital dynamics.

We propose that the relative positions of the Moon and Sun in the sky, which determine the magnitude and variations of the total force of gravity and the lunisolar gravimetric tides, the effects of which are known to regulate several features of plant life [83], might provide a reliable cue of the approaching celestial event. In fact, gravitational forces on Earth are maximal when the Moon is simultaneously close or at its perigee (at the nearest point to the Earth) and syzygy (full or new Moon), which is precisely the planetary configuration we observed on the day of the solar eclipse at the study site. This perigee–syzygy configuration, where the Moon passes exactly the same node at which its orbital plane intersects with the ecliptic Earth–Sun plane, characterizing a certain Saros eclipse series (in our case, Saros 124) occurs every 18 years. Such a time-specific power surge in lunisolar gravimetric forces signalling an eclipse event could generate the anticipatory cue that older trees had experienced during similar events in the past (older trees were, in fact, the most responsive in their anticipation). Considering that gravity is challenging to manipulate experimentally, and the lunisolar gravimetric tides are a product of intricate astronomical configurations that are difficult to simulate, upcoming eclipses offer opportunities to collect data for assessing the potential impact of this gravitational signal.

## 5. Conclusion

Our understanding of collective synchronized behavioural phenomena has advanced significantly through the study of animal groups [76]. Our findings demonstrate that synchronization is equally critical in plant behaviour, revealing its broader relevance across diverse life forms. Furthermore, the thermal state describing the trees and their environment (as defined in equation (S6) in electronic supplementary material, section S5) inherently incorporates Bayes’ probability rule [84], estimating the most likely response to ensure tree survival, conditioned by environmental events.

We propose that, despite the vast differences among organisms, the ability to respond collectively and in accordance with Bayes’ rule to external inputs, changes or perturbations is a shared mechanism across taxa, including plants. These behaviours reflect a broader adaptive strategy for coping with rapid and unpredictable changes, reinforcing ecosystem resilience and emphasizing the significance of synchronized behaviour across species [76].

Thus, recognizing this commonality in synchronized responses across organisms enhances our understanding of biological processes and suggests universal principles governing collective behaviour in both plants and animals. This insight into the fundamental mechanisms of behavioural propagation across life forms offers a new perspective on how ecosystems achieve resilience and adaptability.

## Data Availability

Data are available online [85]. Supplementary material is available online [86].
